# Structural plasticity in the dentate gyrus- revisiting a classic injury model

**DOI:** 10.3389/fncir.2013.00017

**Published:** 2013-02-18

**Authors:** Julia V. Perederiy, Gary L. Westbrook

**Affiliations:** Vollum Institute, Oregon Health and Science UniversityPortland, OR, USA

**Keywords:** perforant path lesion, adult neurogenesis, dentate gyrus, reactive synaptogenesis

## Abstract

The adult brain is in a continuous state of remodeling. This is nowhere more true than in the dentate gyrus, where competing forces such as neurodegeneration and neurogenesis dynamically modify neuronal connectivity, and can occur simultaneously. This plasticity of the adult nervous system is particularly important in the context of traumatic brain injury or deafferentation. In this review, we summarize a classic injury model, lesioning of the perforant path, which removes the main extrahippocampal input to the dentate gyrus. Early studies revealed that in response to deafferentation, axons of remaining fiber systems and dendrites of mature granule cells undergo lamina-specific changes, providing one of the first examples of structural plasticity in the adult brain. Given the increasing role of adult-generated new neurons in the function of the dentate gyrus, we also compare the response of newborn and mature granule cells following lesioning of the perforant path. These studies provide insights not only to plasticity in the dentate gyrus, but also to the response of neural circuits to brain injury.

## Plasticity in the adult brain

The ability of the mammalian brain to change with experience is perhaps its most important feature. At the organismal level, the positive (adaptive) benefits of experience-dependent changes underlie our abilities to learn, speak multiple languages, ride a bicycle and so on. However, equally important are enduring negative (maladaptive) effects that are associated with experience-dependent changes including benign habits as well as more disruptive conditions such as anxiety, post-traumatic stress, and drug addiction. In both cases, these changes are manifested at the level of circuits and individual neurons as a reordering of gene expression profiles, synaptic strength, and circuit connectivity. Reorganization reflects the adaptation of the network to a changing environment, either encoding new information or compensating for injury-induced degeneration. Reorganization following a brain injury inevitably perturbs the dynamic equilibrium, which can affect many aspects of neuronal structure and function including intrinsic neuronal properties, synaptic interactions, and connectivity within and between networks. The cellular and molecular landscape can impose limits on plasticity and regenerative capacity of the adult brain.

A variety of injury models have been used to examine the response of the brain such as crush injuries to peripheral nerves, cortical stab wounds, and spinal cord injury (SCI) models. For example, SCI models have been extensively examined for factors that limit the growth of axons following damage or transection (Akbik et al., 2012; Tuszynski and Steward, 2012). Here we focus on the perforant path lesion, a brain injury model that interrupts the main excitatory input to the dentate gyrus of the hippocampus. This model has the experimental advantages of a highly laminated structure and allows analysis of not only the axonal response to injury, but also changes in dendrite morphology and synaptic reorganization. This classic lesion provided some of the first evidence for structural plasticity following injury in the CNS, and also provides an opportunity to examine the injury response of some of the most highly plastic neurons in the brain, adult-generated newborn granule cells. Because perforant path axons are lesioned at a site remote from the dentate, this model is particularly useful to evaluate axonal sprouting from other pathways terminating in the dentate gyrus. Prior results indicate that axonal sprouting occurs, but only in a lamina-specific manner. There are also compensatory changes in dendritic structure and dendritic spines on the post-synaptic mature granule cells, including an initial reduction in dendritic complexity and spine counts, followed by a limited recovery, presumably based on axonal sprouting. Recent studies with adult-generated granule cells indicate that these cells are highly dynamic following denervation, surprisingly developing dendritic spines in the denervated zone in the absence of functional input. These latter studies suggest that a unique post-lesion environment affects development of dendritic spines and new synapses in deafferented laminae. Before discussing the insights gained from the perforant path lesion model, we first highlight features of neuronal and non-neuronal plasticity that drive adaptive and maladaptive changes in brain circuits.

### Synaptic and dendritic plasticity in the injured brain

**Figure 1:**
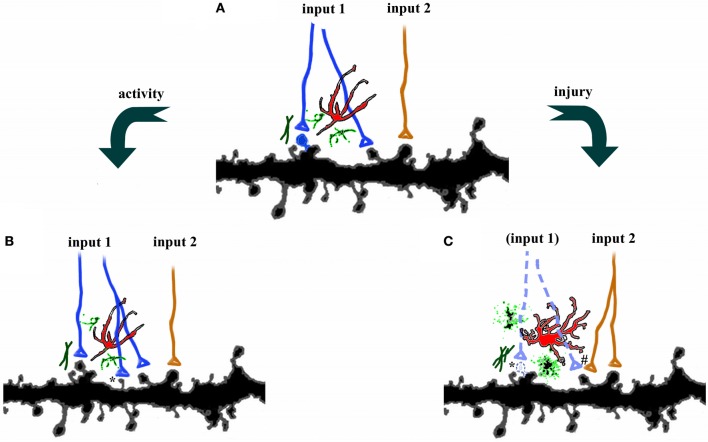
**Plasticity in the central nervous system. (A)** Axons from two different pathways synapse onto spines on the same dendrites. Each synapse is surrounded by astrocytes (red), microglia (green), and extracellular matrix. **(B)** Increases in activity, such as occur during learning, can strengthen connections by axonal sprouting (blue) as well as formation of new filopodia and dendritic spines (^*^). Adjacent afferents, surrounding glia, and extracellular matrix are relatively unaffected. **(C)** Disruption of afferents, such as following injury, leads to degeneration of damaged axons (dotted lines), activation of astrocytes, microglia, and extracellular matrix, as well as retraction of dendritic spines (^*^). Compensatory sprouting of undamaged afferents from another brain region (orange) can form new synapses, including contacts with denervated spines (^#^).

It is well known that synaptic and dendritic plasticity occur in sensory systems following deprivation, and in motor systems following disuse (Hickmott and Steen, 2005; Hofer et al., 2006). However, spines and dendrites also undergo dynamic functional and structural changes following acute injury or neurodegeneration. These changes fall into several categories including retraction of dendritic arbors following loss of inputs, compensatory increases in dendritic arbors in domains of afferent inputs unaffected by the injury, transient changes in spine densities, and alterations in the types or shapes of dendritic spines. For example, dendritic reorganization occurs after ischemia (Hosp and Luft, 2011), but the degree of remodeling depends on the proximity of dendrites to the site of infarction. Brown et al. (2010) reported a dendritic retraction following ischemic injury in cortex adjacent to the infarct, but compensatory dendritic outgrowth away from the site of injury. On the other hand, Mostany and Portera-Cailliau (2011) saw only dendritic pruning at cells in peri-infarct cortex. Dendritic spine density is also sensitive to ischemia (Brown et al., 2008) and SCI (Kim et al., 2006), both of which lead to a reduction in spine density and elongation of the remaining spines, albeit at different time scales. Because spine elongation is associated with synaptogenesis, the underlying mechanisms for these changes are in many cases thought to be sensitive to injury-induced alterations in network activity. For example, the intense neuronal activity associated with kainate-induced seizures triggers beading of dendrites and subsequent loss of spines (Drakew et al., 1996; Zeng et al., 2007). However, brief seizure activity can also trigger more “physiological” responses, such as the induction LTP in CA3 pyramidal neurons (Ben-Ari and Gho, 1988). This dichotomy suggests that network responses to injury are likely to be context-specific, and may reflect exaggerations of the normal adaptive responses to stimuli (Figure 1).

### Sprouting and the axonal response to injury

Axons can also reorganize following injury, although the extent of regeneration varies. In the peripheral nervous system, regenerating axons can grow long distances and re-innervate their targets, thus leading to functional recovery. However, regenerating axons in the central nervous system are often unable to penetrate the lesion, thus limiting long-range axonal outgrowth. Perhaps most extensively studied examples are experimental models of SCI, in which cut or damaged axons of the corticospinal tract form retraction bulbs and eventually move away from the lesion site, unable to penetrate the gliotic scar (Hill et al., 2001; Fitch and Silver, 2008). However, if the transection is incomplete, sprouting of uninjured axons, as well as cortical reorganization can lead to partial functional recovery following injury (Raineteau and Schwab, 2001; Maier and Schwab, 2006). The difference in the capacity for axonal regeneration in the peripheral and central nervous systems reflects differences in intrinsic neuronal properties (Liu et al., 2011) and in post-injury changes in the extracellular environment (Giger et al., 2010). Whereas degenerating material in the peripheral nervous system is effectively cleared following injury (Chen et al., 2007; Bosse, 2012), these processes are much slower in the central nervous system (Vargas and Barres, 2007; Giger et al., 2010), and may thus interfere with reinnervation of deafferented target areas. Axonal structural plasticity may also be maladaptive following injury, as can occur in the brain of patients with temporal lobe epilepsy. Following seizures, mossy fiber axons sprout recurrent collaterals that synapse onto granule cell dendrites in the inner molecular layer, thereby increasing excitatory connectivity within the dentate gyrus (Sutula and Dudek, 2007). Such structural reorganization can lead to an imbalance between excitation and inhibition in the circuit, which may underlie recurrent seizures.

### Glial and extracellular response to brain injury

**Figure 2:**
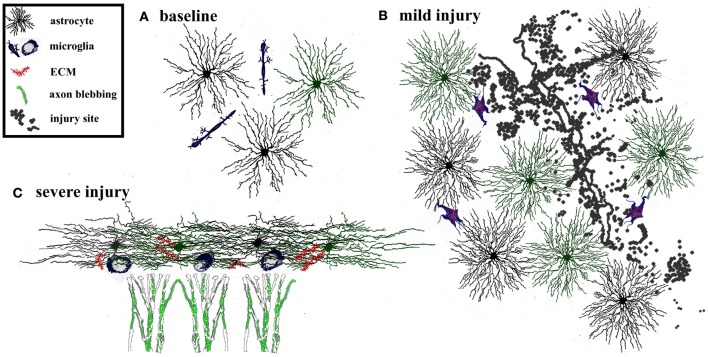
**Adaptive and maladaptive glial changes following injury.** The degree of astrogliosis depends on the severity of injury. **(A)** Glia and extracellular matrix at baseline. Astrocytes are tiled, i.e., their processes do not overlap with neighboring astrocytes. Microglia are interspersed throughout the region. **(B)** Mild injury triggers activation of microglia and astrocytes. Astrocytes and microglia increase in size and acquire more complex process morphology, but astrocytes maintain their tiled formation. This response is considered adaptive because it limits the spread of degeneration away from the site of injury, dampens excitotoxicity, and promotes tissue regeneration. Such glial activation typically resolves within a few weeks after a mild, transient injury. **(C)** In contrast severe injury causes reactive astrocytes to invade neighboring domains, recruit reactive microglia, and increases secretion of extracellular molecules. This results in formation of a persistent glial scar that can be impenetrable to sprouting axons.

Glial cells are intimately involved in function and plasticity of the healthy adult brain, however, their contribution to recovery following injury is even more striking. Brain and spinal cord trauma, neurodegeneration, ischemia, and infection, all stimulate morphological and molecular changes in surrounding astrocytes, often referred to as reactive gliosis. Depending on the triggering mechanism and its duration, the glial response can promote or inhibit recovery (Figure 2; Sofroniew, 2009). For example, during mild insults to the CNS, such as the immune reaction that follows a viral infection or as occurs in areas distant to a lesion site, astrocytes hypertrophy but remain tiled (Figure 2B; Wilhelmsson et al., 2006). In such cases, tissue reorganization is minimal and reactive astrogliosis resolves within a few weeks. However, following more severe CNS insults such as major trauma, stroke, or neurodegeneration, astrocytes proliferate, acquire expansive reactive morphology, and their processes extend beyond their original borders (Sofroniew and Vinters, 2010). The resulting dense network of newly proliferated astrocytes can recruit other cell types, including fibromeningeal cells and microglia, resulting in the formation of a permanent and impenetrable glial scar (Figure 2C). Reactive astrogliosis has traditionally been viewed as maladaptive because gliosis can contribute to glutamate toxicity (Takano et al., 2005), generation of seizures (Jansen et al., 2005; Tian et al., 2005), inflammation (Brambilla et al., 2005), and chronic pain (Milligan and Watkins, 2009). Furthermore, the glial scar can inhibit axonal regrowth (Silver and Miller, 2004). Although experimental interference with glial scar formation can increase axonal regeneration, it can also increase lesion size and diminish functional recovery (Sofroniew, 2009). The latter suggests that the presence of reactive astrocytes, depending on the context, can have positive effects on neuronal reorganization by stabilizing the extracellular ion balance, reducing seizure likelihood, and dampening excitotoxicity (Rothstein et al., 1996; Koistinaho et al., 2004; Swanson et al., 2004).

An important product of glial cells, the extracellular matrix (ECM), surrounds the synapse (Dityatev et al., 2006, 2010b) and is instrumental in synaptic plasticity both in the healthy and injured brain (Dityatev and Fellin, 2008; Dityatev et al., 2010a; Frischknecht and Gundelfinger, 2012). For example, astrocyte-derived ECM components, such as thrombospondins, initiate synaptic development (Christopherson et al., 2005; Xu et al., 2010) as well as regulate synaptic plasticity (Eroglu, 2009). In addition, inactive perisynaptic matrix metalloproteases are transiently activated following induction of LTP in the hippocampus (Nagy et al., 2006; Bozdagi et al., 2007). Because ECM components originate from glia, activation of astrocytes following injury can affect expression of ECM molecules and thus post-injury neuroplasticity. Like the astroglial response, these molecules can have a dual role in recovery. For example, chondroitin sulfate proteoglycan expression is beneficial in containing the size of a lesion, but a few days later can inhibit axonal growth (Zuo et al., 1998; Galtrey and Fawcett, 2007). Likewise, matrix metalloproteinases have a positive effect on reactive synaptogenesis when transiently upregulated (Falo et al., 2006), but persistent and widespread MMP expression leads to regression of dendritic spines, degeneration of synapses and neuronal apoptosis (Falo et al., 2006; Huntley, 2012). The complexity of the glial and ECM response underscores both the potential for, and the limitations of, repair and regeneration following brain injury.

## Perforant path lesion as a model of post-injury plasticity in the adult brain

### Advantages of model

Lesioning of the perforant path was one of the first models to document injury-induced plasticity in the adult brain. This lesion of the major excitatory input into the dentate gyrus affects the trisynaptic hippocampal circuit, disrupting the distinctly unidirectional progression of excitatory activity arriving from other brain regions (Knowles, 1992). Because the entorhinal lesion site is distant from the dentate gyrus, local degenerative/inflammatory effects at the lesion site can be easily separated from the regenerative effects of post-lesion circuit reorganization. The simple cyto- and fiber architecture and lamination pattern of the dentate gyrus also provides an experimental advantage because the lesion affects only one of many afferent fiber systems. Each afferent input terminates in a specific lamina of the molecular layer (Hjorth-Simonsen and Jeune, 1972) and each is functionally and molecularly distinct (Leranth and Hajszan, 2007). This diversity allows a comparison of heterotypic and homotypic sprouting post-lesion (Ramirez, 2001), as the balance of these inputs may have a role in functional recovery.

### Post-lesion circuit reorganization—axons

**Figure 3:**
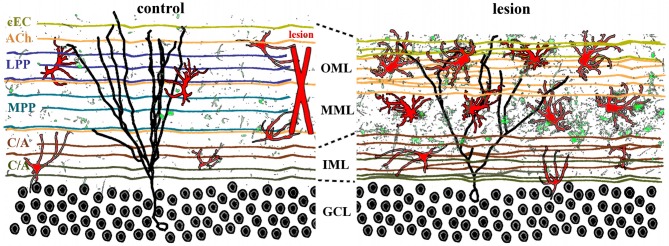
**Lamina-specific axon sprouting and reactive gliosis following perforant path lesion.** The molecular layer of the adult dentate gyrus is a highly laminated structure with afferent inputs segregated based on their origin and neurotransmitter phenotype. All afferent axons form either symmetrical or asymmetrical synapses with mature granule cells (black traces) in a lamina-specific manner. **Left panel**: the inner molecular layer (IML) is occupied by the glutamatergic commissural/associational fibers (C/A) that arise from mossy cells in the ipsi- or contralateral hilus. The middle and outer molecular layer (MML, OML) are occupied predominantly by the glutamatergic perforant path (MPP, LPP), which originates in the ipsilateral entorhinal cortex. In rats (but not in mice), there is also a crossed glutamatergic projection from the contralateral entorhinal cortex (cEC) that terminates in the outermost molecular layer (OML). Cholinergic axons (ACh) from the septal nuclei/diagonal band of Broca are interspersed throughout the molecular layer, as are astrocytes (red) and quiescent microglia (green). **Right panel**: lesion of the entorhinal cortex (red X, left panel) transects both medial and lateral perforant path, thus eliminating the majority of excitatory input into the dentate gyrus. Degeneration of these axons induces lamina-specific sprouting of the remaining septohippocampal (ACh), commissural/associational (C/A), and crossed entorhino-dentate (cEC) afferents. In the rat, the contralateral entorhino-dentate projection (cEC) partially restores excitatory innervation of the mature granule cells (black trace), however, their dendritic length and complexity are still reduced. The microglia (green) and astrocytes (red) become “activated” following lesion, but this response is limited to the deafferented zone. Note the expansion of the inner molecular layer and shrinkage of the outer layers.

Afferents to the dentate gyrus have diverse origins and neurotransmitter phenotypes that converge on the hippocampus (Figure 3, left panel). Glutamatergic inputs to the outer two-thirds of the dentate molecular layer include the entorhinodentate perforant path (Hjorth-Simonsen and Jeune, 1972; van Groen et al., 2003) and a weak species-specific commissural projection from the contralateral entorhinal cortex (van Groen et al., 2002; Deller et al., 2007). Glutamatergic input to the inner molecular layer consists of the mossy cell axons from the commissural/associational (C/A) collaterals (Gottlieb and Cowan, 1973; Soriano and Frotscher, 1994). These excitatory synaptic inputs are complemented by cholinergic, GABAergic, noradrenergic, dopaminergic, and serotonergic projections that terminate throughout the molecular layer (Leranth and Hajszan, 2007). Because the entorhinodentate projection is the largest glutamatergic afferent fiber system, a perforant path lesion severs the majority of excitatory innervation in the dentate gyrus, thus effectively denervating the outer two-thirds of the molecular layer and vacating 80–90% of all synapses in that region (Matthews et al., 1976a; Steward and Vinsant, 1983). Such degeneration of excitatory synapses triggers compensatory axonal sprouting that is lamina-specific (Frotscher et al., 1997) and can be either homo- or heterotypic, depending on the neurotransmitter involved. Sprouting of other glutamatergic axons, defined as homotypic to the perforant path, includes the weak entorhinodentate projection from the contralateral, non-lesioned entorhinal cortex that normally terminates in the deafferented region (Steward et al., 1973; Steward, 1976; Cotman et al., 1977; Deller et al., 1996a), and the glutamatergic component of the commissural/associational fiber system that normally terminates in the inner molecular layer (Gall and Lynch, 1981; Deller et al., 1996b). Although homotypic reactive sprouting can partially replace lost synapses in the denervated zone (Marrone et al., 2004), the degree of excitatory reinnervation is species-specific (van Groen et al., 2002; Del Turco et al., 2003; Deller et al., 2007). Homotypic sprouting also can partially restore postsynaptic function (Reeves and Steward, 1988) as well as ameliorate some behavioral deficits (Ramirez, 2001).

**Figure 4:**
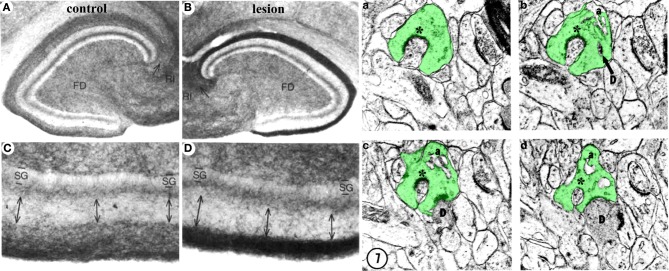
**Structural plasticity following perforant path lesions. Left panels** (modified from Steward and Messenheimer, 1978): Mature cat hippocampus histochemically stained for acetyl cholinesterase (AChE) activity at 60 days post-lesion. The density of AChE is dramatically increased in the denervated outer molecular layer (**A,B**, top right, dark band), consistent with sprouting of the cholinergic septohippocampal axons following lesion. Also note that the thickness of the inner molecular layer is increased due to sprouting of the glutamatergic commissural/associational fibers (**C,D**, bottom right, double arrows). **Right panels** (modified from Matthews et al., 1976b): Ultrastructural evidence for synaptic regeneration in the denervated zone at 60 days post-lesion in the mature rat. Serial sections through a complex spine (a,b,c,d, green) show synaptic contacts with a degenerating bouton “D” as well as with a regenerating axon “^*^.” a = spine apparatus.

Lesion of the perforant path also triggers reactive heterotypic sprouting of non-glutamatergic afferents such as the cholinergic septodentate projection. Sprouting of this fiber system was initially detected as an increase in acetylcholinesterase (AChE) staining in the denervated zone (Figure 4; Lynch et al., 1972; Nadler et al., 1977a,b). The width of the AChE band was subsequently correlated with the extent of the lesion and the time course of reorganization (Zimmer et al., 1986; Steward, 1992), and therefore has been used as a marker for the extent and completeness of a perforant path lesion. Although the increase in AChE staining density in the denervated region has been corroborated (Vuksic et al., 2011), it remains uncertain whether this increase indicates actual cholinergic sprouting or is a consequence of post-lesion tissue shrinkage (Phinney et al., 2004). Perforant path lesions also cause sprouting of GABAergic C/A axons (Deller et al., 1995) as well as trigger receptor reorganization and new inhibitory synapse formation on mature granule cells (Simbürger et al., 2000, 2001). In combination with a decrease in glutamatergic innervation, these results suggest that lesions of the perforant path can alter the excitation/inhibition balance in the dentate gyrus (Clusmann et al., 1994), which can potentially complicate functional recovery. However, heterotypic sprouting may also serve an adaptive purpose in post-lesion circuit reorganization by reinnervating vacated synapses and thus preventing or delaying transsynaptic cell death.

### Post-lesion circuit reorganization—dendrites/spines

Interruption of the perforant path denervates one of the main inputs to the principal neurons in the adult dentate gyrus—the mature granule cells. These cells are part of the trisynaptic hippocampal circuit, with their dendrites receiving afferent input from the entorhinal cortex and other brain regions; and their axons forming the mossy fibers that synapse with pyramidal cells in CA3. The two subdivisions of the perforant path, medial and lateral, synapse with mature granule cell dendrites in the middle and outer molecular layers, respectively (Hjorth-Simonsen and Jeune, 1972; van Groen et al., 2003). Following a perforant path lesion, these axons degenerate (Matthews et al., 1976a), thus eliminating the majority of excitatory input onto dendritic segments in the outer two-thirds of the molecular layer (Figure 3). The loss of excitatory input initiates a series of morphological and functional changes in the post-synaptic mature granule cells. Dendrites retract, resulting in less complex dendritic arbors in the denervated region (Caceres and Steward, 1983; Diekmann et al., 1996; Schauwecker and McNeill, 1996; Vuksic et al., 2011). Distal dendritic segments are progressively lost for periods up to 90 days post-lesion, with some recovery by 180 days post-lesion. However, the recovery most likely reflects the extension of existing dendrites, rather than formation of new branches (Vuksic et al., 2011). Similarly, the density of dendritic spines—the postsynaptic targets of the entorhinodentate projection—is significantly reduced following lesion, but only in the deafferented zone (Parnavelas et al., 1974; Vuksic et al., 2011). Surprisingly there is relatively little data assessing the functional state of the dentate gyrus circuit following such lesions. However, spontaneous neural activity in mature granule cells post-lesion appears to transiently decrease immediately following lesion, then gradually returns to pre-lesion levels by 8 days (Reeves and Steward, 1988). The source of this activity presumably reflects reorganization of synaptic inputs that follows excitatory reinnervation by sprouting afferents.

### Post-lesion glial and extracellular matrix (ECM) response

Post-lesion structural reorganization in the adult dentate gyrus is influenced by the post-injury dynamics of the extracellular environment. Reactive gliosis following perforant path lesion is both rapid and sustained, and is considered adaptive in this context. Gliosis serves to clear degenerating debris, to maintain laminar borders, and to aid reactive synaptogenesis in the deafferented region. For example, microglia proliferate and acquire reactive morphology within 3 days post-lesion and return to baseline by day 10 (Hailer et al., 1999). However, activation of astrocytes in the denervated zone is delayed relative to microglia and persists for at least 30 days post-lesion (Hailer et al., 1999). Together, microglia and astrocytes participate in phagocytosis of degenerating axons (Bechmann and Nitsch, 2000) and may regulate axon sprouting and reactive synaptogenesis (Gage et al., 1988; Ullian et al., 2004). The efficiency of phagocytosis following injury, especially of degenerating myelinated axons, generally correlates with the degree of regeneration in the CNS (Neumann et al., 2009). Because the glial response is limited to the denervated lamina with relatively little reactive gliosis in the inner molecular layer, this lamina-specific reaction may underlie the lack of sprouting across laminar borders into the denervated zone. Reactive gliosis also triggers changes in the extracellular matrix, which may affect the maintenance of laminar borders following lesion. For example, tenascin-C (Deller et al., 1997) and chondroitin sulfate proteoglycans (Haas et al., 1999) are secreted by reactive astrocytes following perforant path lesion. Both these factors affect axon outgrowth during development as well as following injury (Bovolenta and Fernaud-Espinosa, 2000; Bartus et al., 2012). Similarly, reactive astrocytes can secrete thrombospondins or matrix metalloproteases (Christopherson et al., 2005; Warren et al., 2012), which can provide a scaffold for lesion-induced synaptogenesis (Deller et al., 2001; Mayer et al., 2005).

In summary, lesion of the perforant path eliminates the main excitatory input in the outer two-thirds of the dentate molecular layer, thus partially denervating dendrites of mature granule cells. This lesion illustrates both the potential for regeneration in the CNS, but also some of the limits. Within 2 weeks post-lesion, remaining afferent homo- and heterotypic systems can sprout, but the laminar borders largely limit reorganization of axons and synaptic terminals. Changes in the composition of the extracellular matrix, triggered by degenerating perforant path axons and reactive gliosis, are a major contributing factor in this regard.

## Adult-generated newborn neurons and the response to brain injury

Plasticity of neuronal circuits occurs in the adult mammalian brain and is particularly intriguing in the form of adult neurogenesis (Lledo et al., 2006). The dentate gyrus of the hippocampal formation harbors a continuously proliferating population of granule cells precursors, some of which mature over several weeks and become functionally indistinguishable from mature granule cells in the dentate gyrus (van Praag et al., 2002; Overstreet-Wadiche and Westbrook, 2006; Ge et al., 2008). In contrast to mature granule cells, newborn neurons have enhanced synaptic plasticity (Ambrogini et al., 2004; Schmidt-Hieber et al., 2004), suggesting that they may have distinct roles in normal hippocampal function as well as following injury.

### Proliferation of adult-generated neurons following injury

**Figure 5:**
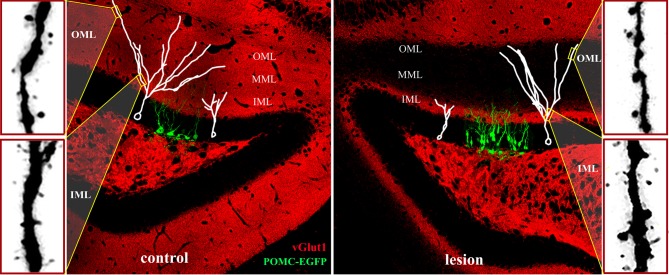
**Adult neurogenesis and synaptic integration following perforant path lesion.** Data montage of dorsal hippocampus in mature mouse. **Left panel:** The morphology of 14-day-old newborn granule cells (POMC-EGFP, green), and typical maturation of newborn granule cells (white traces) shown at 14- (white cell at right in panel) and 21- (white cell at left in panel) days post-mitosis. At 14 days, dendritic arbors are limited to the inner molecular layer (IML) and lack spines, whereas dendrites of 21-day-old granule penetrate the middle (MML) and outer (OML) molecular layers and develop spines. Typical dendritic spine densities are shown at far left for the inner (IML) and outer (OML) molecular layers. **Right panel:** Unilateral perforant path lesion increases proliferation of newborn granule cells (POMC-EGFP, 14 days post-mitosis) and reduces their dendritic outgrowth (white traces). Traces of 14- (left trace) and 21- (right trace) day-old granule cells shown at 14- (left) and 21- (right) days post-lesion, respectively. Dendritic length and complexity are reduced relative to those of newborn granule cells in the contralateral hemisphere (left panel). At 21 days post-lesion *de novo* spine formation in 21-day old granule cells (far right panels) is decreased in the deafferented zone (OML), but increased in the intact inner molecular layer (IML). Note the dramatically reduced staining for a marker for glutamatergic axons (vGlut1, red) at 21 days post-lesion in the middle and outer molecular layers illustrating the absence of excitatory inputs in the denervated zone.

Proliferation of newborn neurons in the dentate gyrus is highly sensitive to environmental and endogenous signals, such as learning, exercise, or severe stress (van Praag et al., 1999; Overstreet et al., 2004; Tashiro et al., 2007). Interestingly, increases in proliferation also occur in various animal models of ischemia, epilepsy, and traumatic brain injury (Liu et al., 1998; Parent, 2003; Jessberger et al., 2005; Lichtenwalner and Parent, 2006; Parent, 2007; Kernie and Parent, 2010). Depending on the stimulus, increased proliferation of neuronal precursors can be adaptive and has therefore been targeted as a potential therapeutic avenue (Magavi et al., 2000; Mitchell et al., 2004; DeCarolis and Eisch, 2010). However, proliferation can also be maladaptive. For example, following seizures, newborn neurons can proliferate and disperse throughout the granule cell layer as well as ectopically in the hilus (Scharfman et al., 2000; Parent et al., 2006; Koyama et al., 2012). Ectopic cells in the hilus show enhanced excitability and fire synchronously with aberrantly reorganized mossy fibers (Scharfman et al., 2000), thus potentially contributing to epileptogenesis (Parent, 2007; Koyama et al., 2012; but see also Buckmaster and Lew, 2011). However, abnormal migration of mature granule cells (without accompanying neurogenesis) has also been reported following seizures induced by intrahippocampal kainic acid (Heinrich et al., 2006), suggesting that both proliferation and dispersion are context-specific. Interestingly proliferation of neuronal precursors is also stimulated by a unilateral lesion of the perforant path, which removes the major input to the dentate gyrus and thus might be expected to reduce neuronal activity in granule cells. A dramatic increase in new granule neurons can be observed in the ipsilateral dentate gyrus at 14 days post-lesion (Figure 5, green cells; Perederiy et al., 2013).

### Dendritic maturation of newborn granule cells

Newly-differentiated neurons in the first 1–2 weeks post-mitosis (Kempermann et al., 2004), have a distinct morphology with small cell bodies, a primary dendrite that is confined to the inner molecular layer, and an immature axon that has reached the CA3 region (Overstreet et al., 2004). Although newborn neurons in the first 1–2 weeks post-mitosis express glutamate receptors, they have yet to make synaptic contact with perforant path axons. Instead, these cells receive depolarizing GABAergic inputs (Ambrogini et al., 2004; Ge et al., 2006), consistent with a trophic role for GABA in neuronal development (Owens and Kriegstein, 2002). Over the subsequent 2 weeks, newborn granule cells extend their dendrites to the middle and outer molecular layers, develop dendritic spines, and are innervated by the glutamatergic perforant path (van Praag et al., 2002; Overstreet-Wadiche and Westbrook, 2006). This stereotyped maturation process provides an ideal opportunity to examine how newborn neurons in the adult dentate gyrus develop in the absence of their main excitatory input from the perforant path. Specifically, one can follow a cohort of new neurons labeled on the day of the injury as they extend processes and form synapses in the weeks following the injury, in this case lesion of the perforant path. As discussed above, this is a dynamic period of extracellular changes and circuit reorganization. At 14 days after a unilateral perforant path lesion—the time of maximal sprouting and reactive synaptogenesis in the deafferented molecular layer—newly developed dendrites on newborn neurons have extended into the intact inner molecular layer. However, their total dendritic length is shorter than dendrites in the contralateral hemisphere (Figure 5, right panel; Perederiy et al., 2013). By 21 days post-lesion, dendrites of 21-day-old neurons have penetrated into the deafferented zone, but the overall dendritic length and complexity are reduced. The dendritic complexity deficit is most pronounced in the distal segments, which at 21 days normally would be contacted by perforant path afferents. The reduced complexity of the dendritic arbor on newborn neuron post-injury is similar in degree to the post-lesion retraction of distal dendritic segments in mature cells (Vuksic et al., 2011).

### Lamina-specific development of dendritic spines following lesion

Although dendritic arbors are reduced in total length and complexity, 21-day-old granule cells in the adult mouse develop dendritic spines in the denervated zone. This is surprising because mice have no detectable entorhino-dentate projection from the contralateral hemisphere (van Groen et al., 2002; Del Turco et al., 2003; Deller et al., 2007). Thus, the spines develop in the apparent absence of functional presynaptic input (Figure 5, red stain/vGlut1). Dendritic spine density, however, is lower than that in the contralateral hemisphere. The newly formed spines in the denervated outer molecular layer have postsynaptic densities, but typically lack a functional apposing presynaptic terminal (Perederiy et al., 2013). What signal substitutes for the presynaptic terminal as these new dendritic spines appear in the denervated zone remains a mystery. One possibility is that the post-lesion environment surrounding distal dendrites provides molecular signals that substitute for glutamatergic axons in the formation of dendritic spines. The overall reduction in spine density in the denervated zone is comparable between dendrites of newborn and mature granule neurons. However, newly formed dendrites in the ipsilateral inner molecular layer show a dramatic increase in spine density relative to those in the contralateral hemisphere (Figure 5, lower far right panel), whereas spine density on mature granule cells is unaffected in this region (Vuksic et al., 2011; Perederiy et al., 2013). The increase in spine density in the inner molecular layer may reflect the enhanced synaptic plasticity of newborn neurons relative to mature granule cells. Immature granule cells in the normal dentate gyrus exhibit decreased LTP induction thresholds at 2–3 weeks and increased LTP amplitudes at 4–6 weeks, which can be observed even with sparse glutamatergic innervation (Schmidt-Hieber et al., 2004; Ge et al., 2007; Lemaire et al., 2012). These observations indicate that newborn neurons are preferentially targeted by sprouting axons in the intact inner molecular layer and suggest that newborn granule cells may be more responsive during circuit reorganization than mature granule cells. Such post-lesion innervation of new dendrites by sprouting homotypic axons may provide a sufficient amount of excitatory input to ensure functional integration and survival of newborn granule cells, thus partially compensating for the degenerated perforant path.

## Limits of plasticity

The perforant path model serves as an example of CNS plasticity that incorporates many features of the injury response. Neuroplasticity in the adult brain is a complex process that involves all aspects of the neural circuit—axonal sprouting and terminal bouton turnover, reorganization of dendrites and spines, activity-dependent modulation of synaptic strength, as well as adult neurogenesis. The dynamic nature of the adult brain gives hope for endogenous repair following injury, however, the limits of neuroplasticity must be recognized in order to optimize medical treatments. Following perforant path lesion, newborn neurons showed a greater degree of structural plasticity than mature granule cells by accommodating sprouting axons in the inner molecular layer. However, circuit-appropriate reinnervation of denervated targets is essential for functional recovery, and this aspect of recovery has yet to be fully explored. For example, following ischemic lesions, newborn neurons from the expanded ipsilateral SVZ can replenish cells lost in the striatum by migrating in chains toward the site of infarction, where they differentiate into medium spiny neurons (Arvidsson et al., 2002; Parent et al., 2002). Interestingly, migration of these cells can persist for at least 1 year after stroke (Kokaia et al., 2006), suggesting that repair mechanisms can remain active long after the insult. Some evidence shows that newly differentiated neurons in the striatum grow dendrites, form synapses, and have spontaneous post-synaptic activity, indicative of functional integration (Hou et al., 2008). However, whether these cells receive appropriate inputs is unknown (Burns et al., 2009). The importance of appropriate reinnervation is perhaps best exemplified by stem cell therapy following SCI. Although promising (Bareyre, 2008; Coutts and Keirstead, 2008), grafting of neural progenitor cells around the lesion site can trigger aberrant axonal sprouting and subsequent pain hypersensitivity in the forepaw (Hofstetter et al., 2005). This issue potentially may be resolved by creating a favorable environment for stem cell maturation and functional integration, including axon guidance molecules, growth factors, and, if necessary, immune suppressors (Liu et al., 2003; Williams and Lavik, 2009). The lamina-specific reorganization following perforant path lesion suggests that effective circuit regeneration and functional recovery will require a rebalancing of the glial response and the extracellular environment, to allow new axons to find their appropriate targets and to provide a permissive scaffold for synaptogenesis.

### Conflict of interest statement

The authors declare that the research was conducted in the absence of any commercial or financial relationships that could be construed as a potential conflict of interest.

## References

[B1] Akbik F. Cafferty W. B. Strittmatter S. M. (2012). Myelin associated inhibitors: a link between injury-induced and experience-dependent plasticity. Exp. Neurol. 235, 43–52 10.1016/j.expneurol.2011.06.006 21699896 PMC3189418

[B2] Ambrogini P. Lattanzi D. Ciuffoli S. Agostini D. Bertini L. Stocchi V. (2004). Morpho-functional characterization of neuronal cells at different stages of maturation in granule cell layer of adult rat dentate gyrus. Brain Res. 1017, 21–31 10.1016/j.brainres.2004.05.039 15261095

[B3] Arvidsson A. Collin T. Kirik D. Kokaia Z. Lindvall O. (2002). Neuronal replacement from endogenous precursors in the adult brain after stroke. Nat. Med. 8, 963–970 10.1038/nm747 12161747

[B4] Bareyre F. M. (2008). Neuronal repair and replacement in spinal cord injury. J. Neurol. Sci. 265, 63–72 10.1016/j.jns.2007.05.004 17568612

[B5] Bartus K. James N. D. Bosch K. D. Bradbury E. J. (2012). Chondroitin sulphate proteoglycans: key modulators of spinal cord and brain plasticity. Exp. Neurol. 235, 5–17 10.1016/j.expneurol.2011.08.008 21871887

[B6] Bechmann I. Nitsch R. (2000). Involvement of non-neuronal cells in entorhinal-hippocampal reorganization following lesions. Ann. N.Y. Acad. Sci. 911, 192–206 10.1111/j.1749-6632.2000.tb06727.x 10911875

[B7] Ben-Ari Y. Gho M. (1988). Long-lasting modification of the synaptic properties of rat CA3 hippocampal neurones induced by kainic acid. J. Physiol. 404, 365–384 2908124 10.1113/jphysiol.1988.sp017294 PMC1190830

[B8] Bosse F. (2012). Extrinsic cellular and molecular mediators of peripheral axonal regeneration. Cell Tissue Res. 349, 5–14 10.1007/s00441-012-1389-5 22476657

[B9] Bovolenta P. Fernaud-Espinosa I. (2000). Nervous system proteoglycans as modulators of neurite outgrowth. Prog. Neurobiol. 61, 113–132 10.1016/S0301-0082(99)00044-1 10704995

[B10] Bozdagi O. Nagy V. Kwei K. T. Huntley G. W. (2007). *In vivo* roles for matrix metalloproteinase-9 in mature hippocampal synaptic physiology and plasticity. J. Neurophysiol. 98, 334–344 10.1152/jn.00202.2007 17493927 PMC4415272

[B11] Brambilla R. Bracchi-Ricard V. Hu W. H. Frydel B. Bramwell A. Karmally S. (2005). Inhibition of astroglial nuclear factor kB reduces inflammation and improves functional recovery after spinal cord injury. J. Exp. Med. 202, 145–156 10.1084/jem.20041918 15998793 PMC2212896

[B12] Brown C. E. Boyd J. D. Murphy T. H. (2010). Longitudinal *in vivo* imaging reveals balanced and branch-specific remodeling of mature cortical pyramidal dendritic arbors after stroke. J. Cereb. Blood Flow Metab. 30, 783–791 10.1038/jcbfm.2009.241 19920846 PMC2949167

[B13] Brown C. E. Wong C. Murphy T. H. (2008). Rapid morphologic plasticity of peri-infarct dendritic spines after focal ischemic stroke. Stroke 39, 1286–1291 10.1161/STROKEAHA.107.498238 18323506

[B14] Buckmaster P. S. Lew F. H. (2011). Rapamycin suppresses mossy fiber sprouting but not seizure frequency in a mouse model of temporal lobe epilepsy. J. Neurosci. 31, 2337–2347 10.1523/JNEUROSCI.4852-10.2011 21307269 PMC3073836

[B15] Burns T. C. Verfaillie C. M. Low W. C. (2009). Stem cells for ischemic brain injury: a critical review. J. Comp. Neurol. 515, 125–144 10.1002/cne.22038 19399885 PMC4112591

[B16] Caceres A. Steward O. (1983). Dendritic reorganization in the denervated dentate gyrus of the rat following entorhinal cortical lesions: a Golgi and electron microscopic analysis. J. Comp. Neurol. 214, 387–403

[B17] Chen Z. L. Yu W. M. Strickland S. (2007). Peripheral regeneration. Annu. Rev. Neurosci. 30, 209–233 10.1146/annurev.neuro.30.051606.094337 17341159

[B18] Christopherson K. S. Ullian E. M. Stokes C. C. Mullowney C. E. Hell J. W. Agah A. (2005). Thrombospondins are astrocyte-secreted proteins that promote CNS synaptogenesis. Cell 120, 421–433 10.1016/j.cell.2004.12.020 15707899

[B19] Clusmann H. Nitsch R. Heinemann U. (1994). Long lasting functional alterations in the rat dentate gyrus following entorhinal cortex lesion: a current source density analysis. Neuroscience 61, 805–815 10.1016/0306-4522(94)90403-0 7838379

[B20] Cotman C. Gentry C. Steward O. (1977). Synaptic replacement in the dentate gyrus after unilateral entorhinal lesion: electron microscopic analysis of the extent of replacement of synapses by the remaining entorhinal cortex. J. Neurocytol. 6, 455–464 894334 10.1007/BF01178228

[B21] Coutts M. Keirstead H. S. (2008). Stem cells for the treatment of spinal cord injury. Exp. Neurol. 209, 368–377 10.1016/j.expneurol.2007.09.002 17950280

[B22] DeCarolis N. A. Eisch A. J. (2010). Hippocampal neurogenesis as a target for the treatment of mental illness: a critical evaluation. Neuropharmacology 58, 884–893 10.1016/j.neuropharm.2009.12.013 20060007 PMC2839019

[B23] Deller T. Del Turco D. Rappert A. Bechmann I. (2007). Structural reorganization of the dentate gyrus following entorhinal denervation: species differences between rat and mouse. Prog. Brain Res. 163, 501–528 10.1016/S0079-6123(07)63027-1 17765735

[B24] Deller T. Frotscher M. Nitsch R. (1995). Morphological evidence for the sprouting of inhibitory commissural fibers in response to the lesion of the excitatory entorhinal input to the rat dentate gyrus. J. Neurosci. 15, 6868–6878 7472444 10.1523/JNEUROSCI.15-10-06868.1995 PMC6578021

[B25] Deller T. Frotscher M. Nitsch R. (1996a). Sprouting of crossed entorhinodentate fibers after a unilateral entorhinal lesion: anterograde tracing of fiber reorganization with *Phaseolus vulgaris*-leucoagglutinin (PHAL). J. Comp. Neurol. 365, 42–55 10.1002/(SICI)1096-9861(19960129)365:1<42::AID-CNE4>3.0.CO;2-J 8821440

[B26] Deller T. Nitsch R. Frotscher M. (1996b). Layer-specific sprouting of commissural fibres to the rat fascia dentata after unilateral entorhinal cortex lesion: a *Phaseolus vulgaris* leucoagglutinin tracing study. Neuroscience 71, 651–660 10.1016/0306-4522(95)00475-0 8867038

[B27] Deller T. Haas C. A. Frotscher M. (2001). Sprouting in the hippocampus after entorhinal cortex lesion is layer- specific but not translaminar: which molecules may be involved? Restor. Neurol. Neurosci. 19, 159–167 12082219

[B28] Deller T. Haas C. A. Naumann T. Joester A. Faissner A. Frotscher M. (1997). Upregulation of astrocyte-derived tenascin-C correlates with neurite outgrowth in the rat dentate gyrus after unilateral entorhinal cortex lesion. Neuroscience 81, 829–846 10.1016/S0306-4522(97)00194-2 9316032

[B29] Del Turco D. Woods A. G. Gebhardt C. Phinney A. L. Jucker M. Frotscher M. (2003). Comparison of commissural sprouting in the mouse and rat fascia dentata after entorhinal cortex lesion. Hippocampus 13, 685–699 10.1002/hipo.10118 12962314

[B30] Diekmann S. Ohm T. G. Nitsch R. (1996). Long-lasting transneuronal changes in rat dentate granule cell dendrites after entorhinal cortex lesion. A combined intracellular injection and electron microscopy study. Brain Pathol. 6, 205–215 8864277 10.1111/j.1750-3639.1996.tb00846.x

[B31] Dityatev A. Fellin T. (2008). Extracellular matrix in plasticity and epileptogenesis. Neuron Glia Biol. 4, 235–247 10.1017/S1740925X09000118 19497143

[B32] Dityatev A. Frischknecht R. Seidenbecher C. I. (2006). Extracellular matrix and synaptic functions. Results Probl. Cell Differ. 43, 69–97 17068968 10.1007/400_025

[B33] Dityatev A. Schachner M. Sonderegger P. (2010a). The dual role of the extracellular matrix in synaptic plasticity and homeostasis. Nat. Rev. Neurosci. 11, 735–746 10.1038/nrn2898 20944663

[B34] Dityatev A. Seidenbecher C. I. Schachner M. (2010b). Compartmentalization from the outside: the extracellular matrix and functional microdomains in the brain. Trends Neurosci. 33, 503–512 10.1016/j.tins.2010.08.003 20832873

[B35] Drakew A. Müller M. Gähwiler B. H. Thompson S. M. Frotscher M. (1996). Spine loss in experimental epilepsy: quantitative light and electron microscopic analysis of intracellularly stained CA3 pyramidal cells in hippocampal slice cultures. Neuroscience 70, 31–45 10.1016/0306-4522(95)00379-W 8848134

[B36] Eroglu C. (2009). The role of astrocyte-secreted matricellular proteins in central nervous system development and function. J. Cell Commun. Signal. 3, 167–176 10.1007/s12079-009-0078-y 19904629 PMC2778595

[B37] Falo M. C. Fillmore H. L. Reeves T. M. Phillips L. L. (2006). Matrix metalloproteinase-3 expression profile differentiates adaptive and maladaptive synaptic plasticity induced by traumatic brain injury. J. Neurosci. Res. 84, 768–781 10.1002/jnr.20986 16862547

[B38] Fitch M. T. Silver J. (2008). CNS injury, glial scars, and inflammation: inhibitory extracellular matrices and regeneration failure. Exp. Neurol. 209, 294–301 10.1016/j.expneurol.2007.05.014 17617407 PMC2268907

[B39] Frischknecht R. Gundelfinger E. (2012). The brain's extracellular matrix and its role in synaptic plasticity. Adv. Exp. Med. Biol. 970, 153–171 10.1007/978-3-7091-0932-8_7 22351055

[B40] Frotscher M. Heimrich B. Deller T. (1997). Sprouting in the hippocampus is layer-specific. Trends Neurosci. 20, 218–223 10.1016/S0166-2236(96)01018-1 9141198

[B41] Gage F. H. Olejniczak P. Armstrong D. M. (1988). Astrocytes are important for sprouting in the septohippocampal circuit. Exp. Neurol. 102, 2–13 10.1016/0014-4886(88)90073-8 3181350

[B42] Gall C. Lynch G. (1981). Fiber architecture of the dentate gyrus following ablation of the entorhinal cortex in rats of different ages: evidence for two forms of axon sprouting in the immature brain. Neuroscience 6, 903–910 10.1016/0306-4522(81)90171-8 7242920

[B43] Galtrey C. M. Fawcett J. W. (2007). The role of chondroitin sulfate proteoglycans in regeneration and plasticity in the central nervous system. Brain Res. Rev. 54, 1–18 10.1016/j.brainresrev.2006.09.006 17222456

[B44] Ge S. Goh E. L. Sailor K. A. Kitabatake Y. Ming G. L. Song H. (2006). GABA regulates synaptic integration of newly generated neurons in the adult brain. Nature 439, 589–593 10.1038/nature04404 16341203 PMC1420640

[B45] Ge S. Sailor K. A. Ming G. L. Song H. (2008). Synaptic integration and plasticity of new neurons in the adult hippocampus. J. Physiol. 586, 3759–3765 10.1113/jphysiol.2008.155655 18499723 PMC2538931

[B46] Ge S. Yang C. H. Hsu K. S. Ming G. L. Song H. (2007). A critical period for enhanced synaptic plasticity in newly generated neurons of the adult brain. Neuron 54, 559–566 10.1016/j.neuron.2007.05.002 17521569 PMC2040308

[B47] Giger R. J. Hollis E. R. I. I. Tuszynski M. H. (2010). Guidance molecules in axon regeneration. Cold Spring Harb. Perspect. Biol. 2:a001867 10.1101/cshperspect.a001867 20519341 PMC2890195

[B48] Gottlieb D. I. Cowan W. M. (1973). Autoradiographic studies of the commissural and ipsilateral association connection of the hippocampus and detentate gyrus of the rat. I. The commissural connections. J. Comp. Neurol. 149, 393–422 10.1002/cne.901490402 4715298

[B49] Haas C. A. Rauch U. Thon N. Merten T. Deller T. (1999). Entorhinal cortex lesion in adult rats induces the expression of the neuronal chondroitin sulfate proteoglycan neurocan in reactive astrocytes. J. Neurosci. 19, 9953–9963 10559403 10.1523/JNEUROSCI.19-22-09953.1999 PMC6782976

[B50] Hailer N. P. Grampp A. Nitsch R. (1999). Proliferation of microglia and astrocytes in the dentate gyrus following entorhinal cortex lesion: a quantitative bromodeoxyuridine-labelling study. Eur. J. Neurosci. 11, 3359–3364 10.1046/j.1460-9568.1999.00808.x 10510203

[B51] Heinrich C. Nitta N. Flubacher A. Müller M. Fahrner A. Kirsch M. (2006). Reelin deficiency and displacement of mature neurons, but not neurogenesis, underlie the formation of granule cell dispersion in the epileptic hippocampus. J. Neurosci. 26, 4701–4713 10.1523/JNEUROSCI.5516-05.2006 16641251 PMC6674063

[B52] Hickmott P. W. Steen P. A. (2005). Large-scale changes in dendritic structure during reorganization of adult somatosensory cortex. Nat. Neurosci. 8, 140–142 10.1038/nn1384 15657598

[B53] Hill C. E. Beattie M. S. Bresnahan J. C. (2001). Degeneration and sprouting of identified descending supraspinal axons after contusive spinal cord injury in the rat. Exp. Neurol. 171, 153–169 10.1006/exnr.2001.7734 11520130

[B54] Hjorth-Simonsen A. Jeune B. (1972). Origin and termination of the hippocampal perforant path in the rat studied by silver impregnation. J. Comp. Neurol. 144, 215–232 10.1002/cne.901440206 4112908

[B55] Hofer S. B. Mrsic-Flogel T. D. Bonhoeffer T. Hübener M. (2006). Lifelong learning: ocular dominance plasticity in mouse visual cortex. Curr. Opin. Neurobiol. 16, 451–459 10.1016/j.conb.2006.06.007 16837188

[B56] Hofstetter C. P. Holmström N. A. Lilja J. A. Schweinhardt P. Hao J. Spenger C. (2005). Allodynia limits the usefulness of intraspinal neural stem cell grafts; directed differentiation improves outcome. Nat. Neurosci. 8, 346–353 10.1038/nn1405 15711542

[B57] Hosp J. A. Luft A. R. (2011). Cortical plasticity during motor learning and recovery after ischemic stroke. Neural Plast. 2011:871296 10.1155/2011/871296 22135758 PMC3202122

[B58] Hou S. W. Wang Y. Q. Xu M. Shen D. H. Wang J. J. Huang F. (2008). Functional integration of newly generated neurons into striatum after cerebral ischemia in the adult rat brain. Stroke 39, 2837–2844 10.1161/STROKEAHA.107.510982 18635857

[B59] Huntley G. W. (2012). Synaptic circuit remodelling by matrix metalloproteinases in health and disease. Nat. Rev. Neurosci. 13, 743–757 10.1038/nrn3320 23047773 PMC4900464

[B60] Jansen L. A. Uhlmann E. J. Crino P. B. Gutmann D. H. Wong M. (2005). Epileptogenesis and reduced inward rectifier potassium current in tuberous sclerosis complex-1-deficient astrocytes. Epilepsia 46, 1871–1880 10.1111/j.1528-1167.2005.00289.x 16393152

[B61] Jessberger S. Römer B. Babu H. Kempermann G. (2005). Seizures induce proliferation and dispersion of doublecortin-positive hippocampal progenitor cells. Exp. Neurol. 196, 342–351 10.1016/j.expneurol.2005.08.010 16168988

[B62] Kempermann G. Jessberger S. Steiner B. Kronenberg G. (2004). Milestones of neuronal development in the adult hippocampus. Trends Neurosci. 27, 447–452 10.1016/j.tins.2004.05.013 15271491

[B63] Kernie S. G. Parent J. M. (2010). Forebrain neurogenesis after focal Ischemic and traumatic brain injury. Neurobiol. Dis. 37, 267–274 10.1016/j.nbd.2009.11.002 19909815 PMC2864918

[B64] Kim B. G. Dai H. N. McAtee M. Vicini S. Bregman B. S. (2006). Remodeling of synaptic structures in the motor cortex following spinal cord injury. Exp. Neurol. 198, 401–415 10.1016/j.expneurol.2005.12.010 16443221

[B65] Knowles W. D. (1992). Normal anatomy and neurophysiology of the hippocampal formation. J. Clin. Neurophysiol. 9, 252–263 1350592

[B66] Koistinaho M. Lin S. Wu X. Esterman M. Koger D. Hanson J. (2004). Apolipoprotein E promotes astrocyte colocalization and degradation of deposited amyloid-beta peptides. Nat. Med. 10, 719–726 10.1038/nm1058 15195085

[B67] Kokaia Z. Thored P. Arvidsson A. Lindvall O. (2006). Regulation of stroke-induced neurogenesis in adult brain—recent scientific progress. Cereb. Cortex 16 Suppl 1, i162–i167 10.1093/cercor/bhj174 16766702

[B68] Koyama R. Tao K. Sasaki T. Ichikawa J. Miyamoto D. Muramatsu R. (2012). GABAergic excitation after febrile seizures induces ectopic granule cells and adult epilepsy. Nat. Med. 18, 1271–1278 10.1038/nm.2850 22797810

[B69] Lemaire V. Tronel S. Montaron M. F. Fabre A. Dugast E. Abrous D. N. (2012). Long-lasting plasticity of hippocampal adult-born neurons. J. Neurosci. 32, 3101–3108 10.1523/JNEUROSCI.4731-11.2012 22378883 PMC6622037

[B70] Leranth C. Hajszan T. (2007). Extrinsic afferent systems to the dentate gyrus. Prog. Brain Res. 163, 63–84 10.1016/S0079-6123(07)63004-0 17765712 PMC1989689

[B71] Lichtenwalner R. J. Parent J. M. (2006). Adult neurogenesis and the ischemic forebrain. J. Cereb. Blood Flow Metab. 26, 1–20 10.1038/sj.jcbfm.9600170 15959458

[B72] Liu C. Y. Apuzzo M. L. Tirrell D. A. (2003). Engineering of the extracellular matrix: working toward neural stem cell programming and neurorestoration–concept and progress report. Neurosurgery 52, 1154–1165 12699561

[B73] Liu J. Solway K. Messing R. O. Sharp F. R. (1998). Increased neurogenesis in the dentate gyrus after transient global ischemia in gerbils. J. Neurosci. 18, 7768–7778 9742147 10.1523/JNEUROSCI.18-19-07768.1998 PMC6793017

[B74] Liu K. Tedeschi A. Park K. K. He Z. (2011). Neuronal intrinsic mechanisms of axon regeneration. Annu. Rev. Neurosci. 34, 131–152 10.1146/annurev-neuro-061010-113723 21438684

[B75] Lledo P. M. Alonso M. Grubb M. S. (2006). Adult neurogenesis and functional plasticity in neuronal circuits. Nat. Rev. Neurosci. 7, 179–193 10.1038/nrn1867 16495940

[B76] Lynch G. Matthews D. A. Mosko S. Parks T. Cotman C. (1972). Induced acetylcholinesterase-rich layer in rat dentate gyrus following entorhinal lesions. Brain Res. 42, 311–318 10.1016/0006-8993(72)90533-1 4115093

[B77] Magavi S. S. Leavitt B. R. Macklis J. D. (2000). Induction of neurogenesis in the neocortex of adult mice. Nature 405, 951–955 10.1038/35016083 10879536

[B78] Maier I. C. Schwab M. E. (2006). Sprouting, regeneration and circuit formation in the injured spinal cord: factors and activity. Philos. Trans. R. Soc. Lond. B Biol. Sci. 361, 1611–1634 10.1098/rstb.2006.1890 16939978 PMC1664674

[B79] Marrone D. F. LeBoutillier J. C. Petit T. L. (2004). Comparative analyses of synaptic densities during reactive synaptogenesis in the rat dentate gyrus. Brain Res. 996, 19–30 10.1016/j.brainres.2003.09.073 14670627

[B80] Matthews D. A. Cotman C. Lynch G. (1976a). An electron microscopic study of lesion-induced synaptogenesis in the dentate gyrus of the adult rat. I. Magnitude and time course of degeneration. Brain Res. 115, 1–21 10.1016/0006-8993(76)90819-2 974734

[B81] Matthews D. A. Cotman C. Lynch G. (1976b). An electron microscopic study of lesion-induced synaptogenesis in the dentate gyrus of the adult rat. II. Reappearance of morphologically normal synaptic contacts. Brain Res. 115, 23–41 10.1016/0006-8993(76)90820-9 974742

[B82] Mayer J. Hamel M. G. Gottschall P. E. (2005). Evidence for proteolytic cleavage of brevican by the ADAMTSs in the dentate gyrus after excitotoxic lesion of the mouse entorhinal cortex. BMC Neurosci. 6:52 10.1186/1471-2202-6-52 16122387 PMC1199600

[B83] Milligan E. D. Watkins L. R. (2009). Pathological and protective roles of glia in chronic pain. Nat. Rev. Neurosci. 10, 23–36 10.1038/nrn2533 19096368 PMC2752436

[B84] Mitchell B. D. Emsley J. G. Magavi S. S. Arlotta P. Macklis J. D. (2004). Constitutive and induced neurogenesis in the adult mammalian brain: manipulation of endogenous precursors toward CNS repair. Dev. Neurosci. 26, 101–117 10.1159/000082131 15711054

[B85] Mostany R. Portera-Cailliau C. (2011). Absence of large-scale dendritic plasticity of layer 5 pyramidal neurons in peri-infarct cortex. J. Neurosci. 31, 1734–1738 10.1523/JNEUROSCI.4386-10.2011 21289182 PMC6623721

[B86] Nadler J. V. Cotman C. W. Lynch G. S. (1977a). Histochemical evidence of altered development of cholinergic fibers in the rat dentate gyrus following lesions. I. Time course after complete unilateral entorhinal lesion at various ages. J. Comp. Neurol. 171, 561–587 10.1002/cne.901710409 833358

[B87] Nadler J. V. Cotman C. W. Paoletti C. Lynch G. S. (1977b). Histochemical evidence of altered development of cholinergic fibers in the rat dentate gyrus following lesions. II. Effects of partial entorhinal and simultaneous multiple lesions. J. Comp. Neurol. 171, 589–604 10.1002/cne.901710410 833359

[B88] Nagy V. Bozdagi O. Matynia A. Balcerzyk M. Okulski P. Dzwonek J. (2006). Matrix metalloproteinase-9 is required for hippocampal late-phase long-term potentiation and memory. J. Neurosci. 26, 1923–1934 10.1523/JNEUROSCI.4359-05.2006 16481424 PMC4428329

[B89] Neumann H. Kotter M. R. Franklin R. J. (2009). Debris clearance by microglia: an essential link between degeneration and regeneration. Brain 132, 288–295 10.1093/brain/awn109 18567623 PMC2640215

[B90] Overstreet L. S. Hentges S. T. Bumaschny V. F. de Souza F. S. Smart J. L. Santangelo A. M. (2004). A transgenic marker for newly born granule cells in dentate gyrus. J. Neurosci. 24, 3251–3259 10.1523/JNEUROSCI.5173-03.2004 15056704 PMC6730035

[B91] Overstreet-Wadiche L. S. Westbrook G. L. (2006). Functional maturation of adult-generated granule cells. Hippocampus 16, 208–215 10.1002/hipo.20152 16411232

[B92] Owens D. F. Kriegstein A. R. (2002). Is there more to GABA than synaptic inhibition? Nat. Rev. Neurosci. 3, 715–727 10.1038/nrn919 12209120

[B93] Parent J. M. (2003). Injury-induced neurogenesis in the adult mammalian brain. Neuroscientist 9, 261–272 10.1177/1073858403252680 12934709

[B94] Parent J. M. (2007). Adult neurogenesis in the intact and epileptic dentate gyrus. Prog. Brain Res. 163, 529–540 10.1016/S0079-6123(07)63028-3 17765736

[B95] Parent J. M. Elliott R. C. Pleasure S. J. Barbaro N. M. Lowenstein D. H. (2006). Aberrant seizure-induced neurogenesis in experimental temporal lobe epilepsy. Ann. Neurol. 59, 81–91 10.1002/ana.20699 16261566

[B96] Parent J. M. Vexler Z. S. Gong C. Derugin N. Ferriero D. M. (2002). Rat forebrain neurogenesis and striatal neuron replacement after focal stroke. Ann. Neurol. 52, 802–813 10.1002/ana.10393 12447935

[B97] Parnavelas J. G. Lynch G. Brecha N. Cotman C. W. Globus A. (1974). Spine loss and regrowth in hippocampus following deafferentation. Nature 248, 71–73 4818565 10.1038/248071a0

[B98] Perederiy J. V. Luikart B. W. Washburn E. K. Schnell E. Westbrook G. L. (2013). Neural injury alters proliferation and integration of adult-generated neurons in the dentate gyrus. J. Neurosci. (in press). 10.1523/JNEUROSCI.4785-12.2013 PMC3692360 23486947

[B99] Phinney A. L. Calhoun M. E. Woods A. G. Deller T. Jucker M. (2004). Stereological analysis of the reorganization of the dentate gyrus following entorhinal cortex lesion in mice. Eur. J. Neurosci. 19, 1731–1740 10.1111/j.1460-9568.2004.03280.x 15078547

[B100] Raineteau O. Schwab M. E. (2001). Plasticity of motor systems after incomplete spinal cord injury. Nat. Rev. Neurosci. 2, 263–273 10.1038/35067570 11283749

[B101] Ramirez J. J. (2001). The role of axonal sprouting in functional reorganization after CNS injury: lessons from the hippocampal formation. Restor. Neurol. Neurosci. 19, 237–262 12082224

[B102] Reeves T. M. Steward O. (1988). Changes in the firing properties of neurons in the dentate gyrus with denervation and reinnervation: implications for behavioral recovery. Exp. Neurol. 102, 37–49 10.1016/0014-4886(88)90076-3 3181351

[B103] Rothstein J. D. Dykes-Hoberg M. Pardo C. A. Bristol L. A. Jin L. Kunci R. W. (1996). Knockout of glutamate transporters reveals a major role for astroglial transport in excitotoxicity and clearance of glutamate. Neuron 16, 675–686 10.1016/S0896-6273(00)80086-0 8785064

[B104] Scharfman H. E. Goodman J. H. Sollas A. L. (2000). Granule-like neurons at the hilar/CA3 border after status epilepticus and their synchrony with area CA3 pyramidal cells: functional implications of seizure-induced neurogenesis. J. Neurosci. 20, 6144–6158 10934264 10.1523/JNEUROSCI.20-16-06144.2000 PMC6772593

[B105] Schauwecker P. E. McNeill T. H. (1996). Dendritic remodeling of dentate granule cells following a combined entorhinal cortex/fimbria fornix lesion. Exp. Neurol. 141, 145–153 10.1006/exnr.1996.0148 8797677

[B106] Schmidt-Hieber C. Jonas P. Bischofberger J. (2004). Enhanced synaptic plasticity in newly generated granule cells of the adult hippocampus. Nature 429, 184–187 10.1038/nature02553 15107864

[B107] Silver J. Miller J. H. (2004). Regeneration beyond the glial scar. Nat. Rev. Neurosci. 5, 146–156 10.1038/nrn1326 14735117

[B108] Simbürger E. Plaschke M. Fritschy J. M. Nitsch R. (2001). Localization of two major GABA(A) receptor subunits in the dentate gyrus of the rat and cell type-specific up-regulation following entorhinal cortex lesion. Neuroscience 102, 789–803 10.1016/S0306-4522(00)00505-4 11182243

[B109] Simbürger E. Plaschke M. Kirsch J. Nitsch R. (2000). Distribution of the receptor-anchoring protein gephyrin in the rat dentate gyrus and changes following entorhinal cortex lesion. Cereb. Cortex 10, 422–432 10.1093/cercor/10.4.422 10769252

[B110] Sofroniew M. V. (2009). Molecular dissection of reactive astrogliosis and glial scar formation. Trends Neurosci. 32, 638–647 10.1016/j.tins.2009.08.002 19782411 PMC2787735

[B111] Sofroniew M. V. Vinters H. V. (2010). Astrocytes: biology and pathology. Acta Neuropathol. 119, 7–35 10.1007/s00401-009-0619-8 20012068 PMC2799634

[B112] Soriano E. Frotscher M. (1994). Mossy cells of the rat fascia dentata are glutamate-immunoreactive. Hippocampus 4, 65–69 10.1002/hipo.450040108 7914798

[B113] Steward O. (1976). Reinnervation of dentate gyrus by homologous afferents following entorhinal cortical lesions in adult rats. Science 194, 426–428 10.1126/science.982024 982024

[B114] Steward O. (1992). Signals that induce sprouting in the central nervous system: sprouting is delayed in a strain of mouse exhibiting delayed axonal degeneration. Exp. Neurol. 118, 340–351 1284863 10.1016/0014-4886(92)90192-s

[B117] Steward O. Cotman C. W. Lynch G. S. (1973). Re-establishment of electrophysiologically functional entorhinal cortical input to the dentate gyrus deafferented by ipsilateral entorhinal lesions: innervation by the contralateral entorhinal cortex. Exp. Brain Res. 18, 396–414 4778785 10.1007/BF00239108

[B115] Steward O. Messenheimer J. A. (1978). Histochemical evidence for a postlesion reorganization of cholinergic afferents in the hippocampal formation of the mature cat. J. Comp. Neurol. 178, 697–710 10.1002/cne.901780407 632377

[B116] Steward O. Vinsant S. L. (1983). The process of reinnervation in the dentate gyrus of the adult rat: a quantitative electron microscopic analysis of terminal proliferation and reactive synaptogenesis. J. Comp. Neurol. 214, 370–386

[B118] Sutula T. P. Dudek F. E. (2007). Unmasking recurrent excitation generated by mossy fiber sprouting in the epileptic dentate gyrus: an emergent property of a complex system. Prog. Brain Res. 163, 541–563 10.1016/S0079-6123(07)63029-5 17765737

[B119] Swanson R. A. Ying W. Kauppinen T. M. (2004). Astrocyte influences on ischemic neuronal death. Curr. Mol. Med. 4, 193–205 15032713 10.2174/1566524043479185

[B120] Takano T. Kang J. Jaiswal J. K. Simon S. M. Lin J. H. Yu Y. (2005). Receptor-mediated glutamate release from volume sensitive channels in astrocytes. Proc. Natl. Acad. Sci. U.S.A. 102, 16466–16471 10.1073/pnas.0506382102 16254051 PMC1283436

[B121] Tashiro A. Makino H. Gage F. H. (2007). Experience-specific functional modification of the dentate gyrus through adult neurogenesis: a critical period during an immature stage. J. Neurosci. 27, 3252–3259 10.1523/JNEUROSCI.4941-06.2007 17376985 PMC6672473

[B122] Tian G. F. Azmi H. Takano T. Xu Q. Peng W. Lin J. (2005). An astrocytic basis of epilepsy. Nat. Med. 11, 973–981 10.1038/nm1277 16116433 PMC1850946

[B123] Tuszynski M. H. Steward O. (2012). Concepts and methods for the study of axonal regeneration in the CNS. Neuron 74, 777–791 10.1016/j.neuron.2012.05.006 22681683 PMC3387806

[B124] Ullian E. M. Christopherson K. S. Barres B. A. (2004). Role for glia in synaptogenesis. Glia 47, 209–216 10.1002/glia.20082 15252809

[B125] van Groen T. Kadish I. Wyss J. M. (2002). Species differences in the projections from the entorhinal cortex to the hippocampus. Brain Res. Bull. 57, 553–556 11923027 10.1016/s0361-9230(01)00683-9

[B126] van Groen T. Miettinen P. Kadish I. (2003). The entorhinal cortex of the mouse: organization of the projection to the hippocampal formation. Hippocampus 13, 133–149 10.1002/hipo.10037 12625464

[B127] van Praag H. Kempermann G. Gage F. H. (1999). Running increases cell proliferation and neurogenesis in the adult mouse dentate gyrus. Nat. Neurosci. 2, 266–270 10.1038/6368 10195220

[B128] van Praag H. Schinder A. F. Christie B. R. Toni N. Palmer T. D. Gage F. H. (2002). Functional neurogenesis in the adult hippocampus. Nature 415, 1030–1034 10.1038/4151030a 11875571 PMC9284568

[B129] Vargas M. E. Barres B. A. (2007). Why is Wallerian degeneration in the CNS so slow? Annu. Rev. Neurosci. 30, 153–179 10.1146/annurev.neuro.30.051606.094354 17506644

[B130] Vuksic M. Del Turco D. Vlachos A. Schuldt G. Müller C. M. Schneider G. (2011). Unilateral entorhinal denervation leads to long-lasting dendritic alterations of mouse hippocampal granule cells. Exp. Neurol. 230, 176–185 10.1016/j.expneurol.2011.04.011 21536031

[B131] Warren K. M. Reeves T. M. Phillips L. L. (2012). MT5-MMP, ADAM-10, and N-cadherin act in concert to facilitate synapse reorganization after traumatic brain injury. J. Neurotrauma. 29, 1922–1940 10.1089/neu.2012.2383 22489706 PMC3390984

[B132] Wilhelmsson U. Bushong E. A. Price D. L. Smarr B. L. Phung V. Terada M. (2006). Redefining the concept of reactive astrocytes as cells that remain within their unique domains upon reaction to injury. Proc. Natl. Acad. Sci. U.S.A. 103, 17513–17518 10.1073/pnas.0602841103 17090684 PMC1859960

[B133] Williams C. A. Lavik E. B. (2009). Engineering the CNS stem cell microenvironment. Regen. Med. 4, 865–877 10.2217/rme.09.62 19903005 PMC2884372

[B134] Xu J. Xiao N. Xia J. (2010). Thrombospondin 1 accelerates synaptogenesis in hippocampal neurons through neuroligin 1. Nat. Neurosci. 13, 22–24 10.1038/nn.2459 19915562

[B135] Zeng L. H. Xu L. Rensing N. R. Sinatra P. M. Rothman S. M. Wong M. (2007). Kainate seizures cause acute dendritic injury and actin depolymerization *in vivo*. J. Neurosci. 27, 11604–11613 10.1523/JNEUROSCI.0983-07.2007 17959803 PMC3934634

[B136] Zimmer J. Laurberg S. Sunde N. (1986). Non-cholinergic afferents determine the distribution of the cholinergic septohippocampal projection: a study of the AChE staining pattern in the rat fascia dentata and hippocampus after lesions, X-irradiation, and intracerebral grafting. Exp. Brain Res. 64, 158–168 3770108 10.1007/BF00238212

[B137] Zuo J. Hernandez Y. J. Muir D. (1998). Chondroitin sulfate proteoglycan with neurite-inhibiting activity is up-regulated following peripheral nerve injury. J. Neurobiol. 34, 41–54 10.1002/(SICI)1097-4695(199801)34:1<41::AID-NEU4>3.0.CO;2-C 9469617

